# Spin-label Order Parameter Calibrations for Slow Motion

**DOI:** 10.1007/s00723-017-0940-7

**Published:** 2017-09-09

**Authors:** Derek Marsh

**Affiliations:** 1 Max-Planck-Institut für biophysikalische Chemie, 37070 Göttingen, Germany; 2 MEMPHYS-Centre for Biomembrane Physics, University of Southern Denmark, Campusvej 55, 5230 Odense M, Denmark

## Abstract

Calibrations are given to extract orientation order parameters from pseudo-powder electron paramagnetic resonance line shapes of ^14^N-nitroxide spin labels undergoing slow rotational diffusion. The nitroxide *z*-axis is assumed parallel to the long molecular axis. Stochastic-Liouville simulations of slow-motion 9.4-GHz spectra for molecular ordering with a Maier–Saupe orientation potential reveal a linear dependence of the splittings, $2A_{\hbox{max} }$ and $2A_{\hbox{min} }$, of the outer and inner peaks on order parameter $S_{zz}$ that depends on the diffusion coefficient $D_{{{\text{R}} \bot }}$ which characterizes fluctuations of the long molecular axis. This results in empirical expressions for order parameter and isotropic hyperfine coupling: $S_{zz} = s_{1} \times \left( {A_{\hbox{max} } - A_{\hbox{min} } } \right) - s_{o}$ and $a_{o}^{{}} = \tfrac{1}{3}\left( {f_{\hbox{max} } A_{\hbox{max} } + f_{\hbox{min} } A_{\hbox{min} } } \right) + \delta a_{o}$, respectively. Values of the calibration constants $s_{1}$, $s_{\text{o}}$, $f_{\hbox{max} }$, $f_{\hbox{min} }$ and $\delta a_{o}$ are given for different values of $D_{{{\text{R}} \bot }}$ in fast and slow motional regimes. The calibrations are relatively insensitive to anisotropy of rotational diffusion $(D_{{{\text{R}}//}} \ge D_{{{\text{R}} \bot }} )$, and corrections are less significant for the isotropic hyperfine coupling than for the order parameter.

## Introduction

Spin-labelled molecules in partially ordered environments are restricted in their rotational amplitude. This is a common situation in liquid crystals or lipid membranes; also for spin labels anchored to a macromolecule, and in ligand binding sites or occlusion cavities. The degree of ordering is expressed by an order parameter that is related to the rotational amplitude and can be determined directly from the electron paramagnetic resonance (EPR) spectrum.

When the nitroxide *z*-axis lies close to the long molecular axis of a spin-labelled molecule, we can extract orientation order parameters from the EPR powder patterns of non-aligned samples, because spectral anisotropy is then largest [1–3]. Notable practical examples are lipid chains incorporating the DOXYL nitroxide (see e.g., [4–6]), and helical peptides containing the TOAC nitroxide residue [7–9]. Such situations also arise frequently in site-directed spin-labelling with MTSSL [10–12], and also for rigidly spin-labelled DNA bases [13].

For rotational motion that is fast relative to the spectral anisotropies, the ^14^N hyperfine tensor ${\mathbf{A}} \equiv \left( {A_{xx} ,A_{yy} ,A_{zz} } \right)$ is partially averaged to give axial principal values:

1 $$\left\langle {A_{//} } \right\rangle = a_{o} + \tfrac{2}{3}\Delta A.S_{zz}$$

2 $$\left\langle {A_{ \bot } } \right\rangle = a_{o} - \tfrac{1}{3}\Delta A.S_{zz}$$

where $a_{o} = \tfrac{1}{3}\left( {A_{xx} + A_{yy} + A_{zz} } \right)$ and $\Delta A = A_{zz} - \tfrac{1}{2}\left( {A_{xx} + A_{yy} } \right)$. The order parameter is given by:

3 $$S_{zz} = \frac{{\left\langle {A_{//} } \right\rangle - \left\langle {A_{ \bot } } \right\rangle }}{\Delta A} \equiv \tfrac{1}{2}\left( {3\left\langle {\cos^{2} \theta_{z} } \right\rangle - 1} \right)$$

where $\theta_{z}$ is the instantaneous angle that the nitroxide *z*-axis makes with the director for ordering, and angular brackets indicate a time or ensemble average over the orientational distribution in $\theta_{z}$.

Values for the order parameter are obtained from the splitting $2A_{\hbox{max} }$ of the outer peaks in an experimental powder pattern, and from that of the inner peaks $2A_{\hbox{min} }$. These peaks in the powder spectrum correspond to turning points in the angular dependence of spectra from macroscopically aligned samples. For fast motion, $A_{\hbox{max} }$ is rigorously equal to $\left\langle {A_{//} } \right\rangle$ [1, 14]. But to obtain $\left\langle {A_{ \bot } } \right\rangle$ we must make a correction to $A_{\hbox{min} }$, because of spectral overlap in the central region of the powder pattern [1, 2, 15, 16]. When the motion is slow on the nitroxide EPR timescale, however, both outer and inner peaks shift relative to the positions specified by the motionally averaged tensor elements $\left\langle {A_{//} } \right\rangle$ and $\left\langle {A_{ \bot } } \right\rangle$. We expect this because slow motion causes shifts in the outer extrema of the powder pattern, even for unrestricted isotropic rotational diffusion [17, 18]. The main purpose here is to obtain appropriate corrections when the spectra are in the slow-motion regime for nitroxide EPR.

## Results

**Fig. 1:**
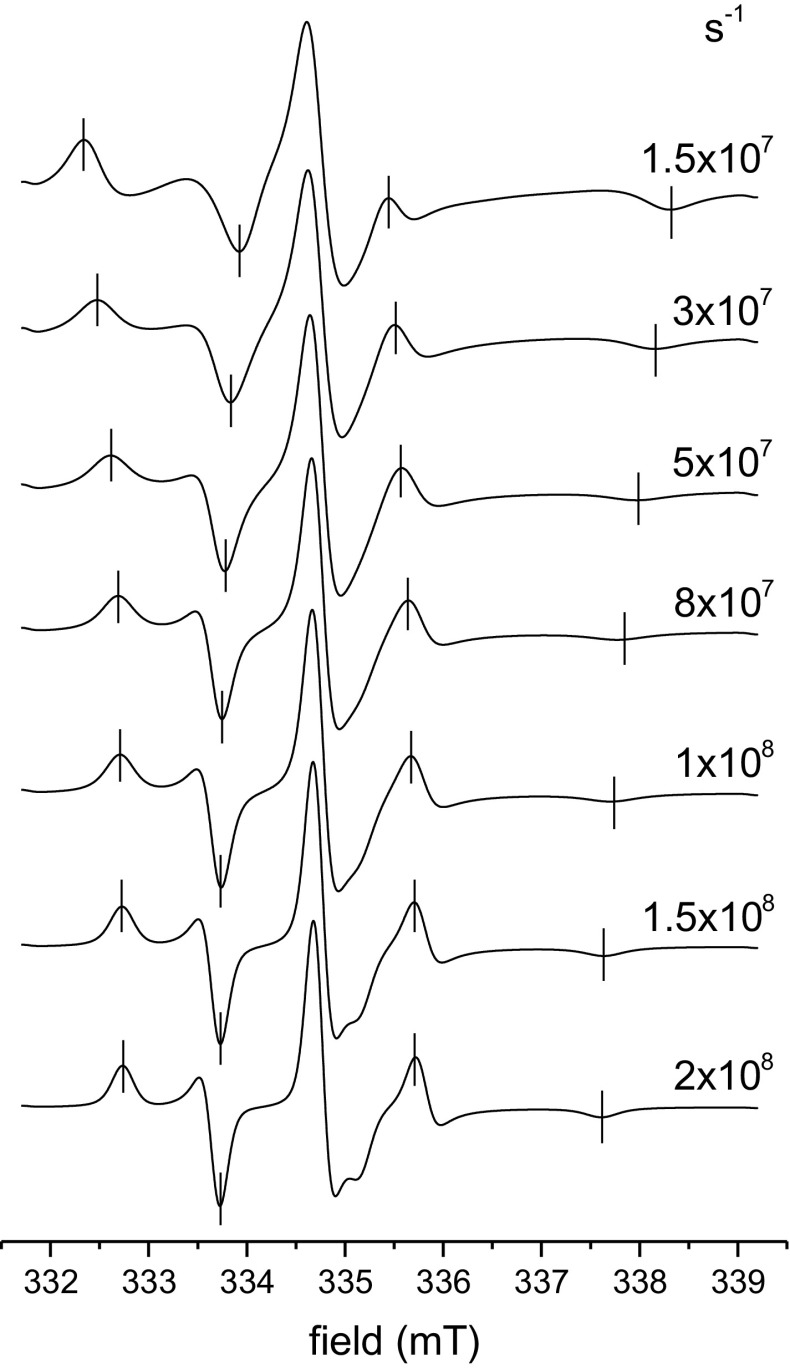
Stochastic-Liouville simulations of ^14^N-nitroxide 9.4-GHz EPR powder spectra, for different values of the rotational diffusion coefficient $D_{{{\text{R}} \bot }} = 0.1D_{{{\text{R}}//}} = 1.5 - 20 \, \times 10^{7} {\text{ s}}^{ - 1}$, as indicated, and fixed order parameter $S_{zz} = 0.5$. Vertical ticks show the positions of the outer and inner hyperfine peaks, which have splittings $2A_{\hbox{max} }$ and $2A_{\hbox{min} }$ respectively. Hyperfine and *g*-tensors are: $(A_{xx} ,A_{yy} ,A_{zz} ) = (0.64,{ 0} . 5 7 , { 3} . 3 3 ) {\text{ mT}}$ and $(g_{xx} ,g_{yy} ,g_{zz} ) = (2.0086,{ 2} . 0 0 5 8 , { 2} . 0 0 2 1 ) { }$. Residual Gaussian broadening, FWHM = 0.18 mT

Figure 1 shows 9.4-GHz EPR spectra for a ^14^N-nitroxide with fixed order parameter $(S_{zz} = 0. 5 )$, and different values of the rotational diffusion coefficient $D_{R \bot }$ that extend from the slow $( \le 1.10^{8} {\text{ s}}^{ - 1} )$ to fast motional regimes. Powder spectra from non-aligned samples are simulated with the stochastic-Liouville equation for Brownian diffusion [19], as implemented in EasySpin 5.1.9 [20], with molecular ordering specified by a Maier–Saupe orientational potential: ${{U(\theta_{z} )} \mathord{\left/ {\vphantom {{U(\theta_{z} )} {k_{\text{B}} T}}} \right. \kern-0pt} {k_{\text{B}} T}} = - \lambda \cos^{2} \theta_{z}$ [21]. In terms of the latter, the order parameter becomes:

4 $$S_{zz} = \frac{3\exp (\lambda )}{{2\lambda \int_{ - 1}^{1} {\exp \left( {\lambda c^{2} } \right)} {\text{d}}c}} - \frac{3}{4\lambda } - \frac{1}{2}$$

from the Boltzmann distribution, where $c \equiv \cos \theta_{z}$. The nitroxide *z*-axis lies along the long molecular axis. The diffusion coefficient for excursions of the molecular long axis (and simultaneously the nitroxide *z*-axis) is $D_{{{\text{R}} \bot }}$, and that for rotation about the same axis $D_{{{\text{R}}//}}$ is ten times faster. As $D_{{{\text{R}} \bot }}$ decreases below $1.10^{8} {\text{ s}}^{ - 1}$, the splitting of the outer peaks in the powder pattern $2A_{\hbox{max} }$ increases and that of the inner peaks $2A_{\hbox{min} }$ decreases, as expected in the slow-motion region. The spectral line shapes also give us some guide as to whether a particular experimental powder pattern contains slow-motional components.

**Fig. 2:**
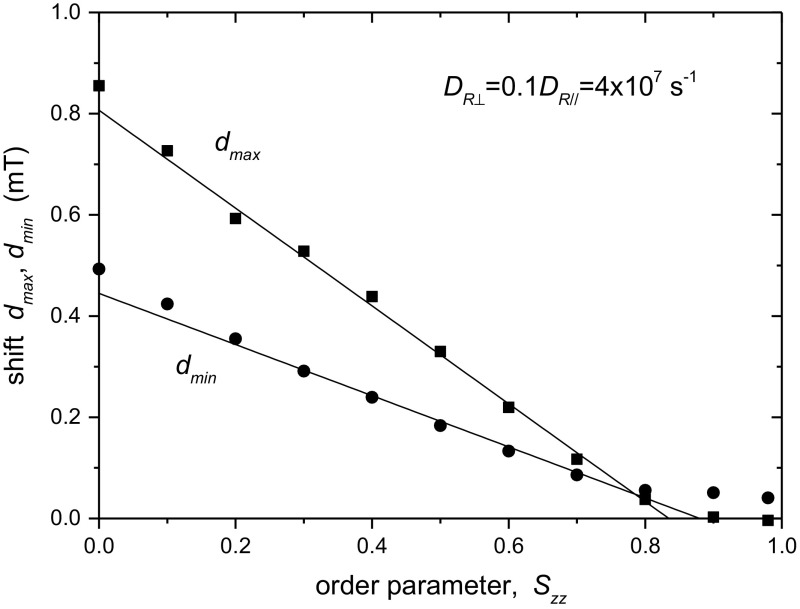
Dependence on order parameter $S_{zz}$ of the shifts, $d_{\hbox{max} }$ (*squares*) and $d_{\hbox{min} }$ (*circles*), in $A_{\hbox{max} }$ and $A_{\hbox{min} }$ from their motionally averaged values $\left\langle {A_{//} } \right\rangle$ and $\left\langle {A_{ \bot } } \right\rangle$, because of slow motion and spectral overlap. Spectra are simulated as in Fig. 1, using a Maier–Saupe orientational potential, and fixed diffusion coefficient $D_{{{\text{R}} \bot }} = 0.1D_{{{\text{R}}//}} = 4\times 10^{7} {\text{ s}}^{ - 1}$. *Straight lines* are linear regressions over the range $S_{zz} = 0.2 - 0.8$ (see Eqs. 5, 6)

In the fast regime, the motionally averaged hyperfine constants $\left\langle {A_{//} } \right\rangle$ and $\left\langle {A_{ \bot } } \right\rangle$ depend linearly on the order parameter according to Eqs. 1 and 2. So also do the experimental constants $A_{\hbox{max} }$ and $A_{\hbox{min} }$. In the slow-motion regime, we find from simulations such as those in Fig. 1 that $A_{\hbox{max} }$ and $A_{\hbox{min} }$ depend approximately linearly on order parameter over the range $S_{zz} = 0. 2- 0. 8$ (see Fig. 2):

5 $$A_{\hbox{max} } = \left\langle {A_{//} } \right\rangle + d_{\hbox{max} }^{\text{o}} \, - d^{\prime}_{\hbox{max} } \times S_{zz}$$

6 $$A_{\hbox{min} } = \left\langle {A_{ \bot } } \right\rangle - d_{\hbox{min} }^{\text{o}} + d^{\prime}_{\hbox{min} } \times S_{zz}.$$

**Fig. 3:**
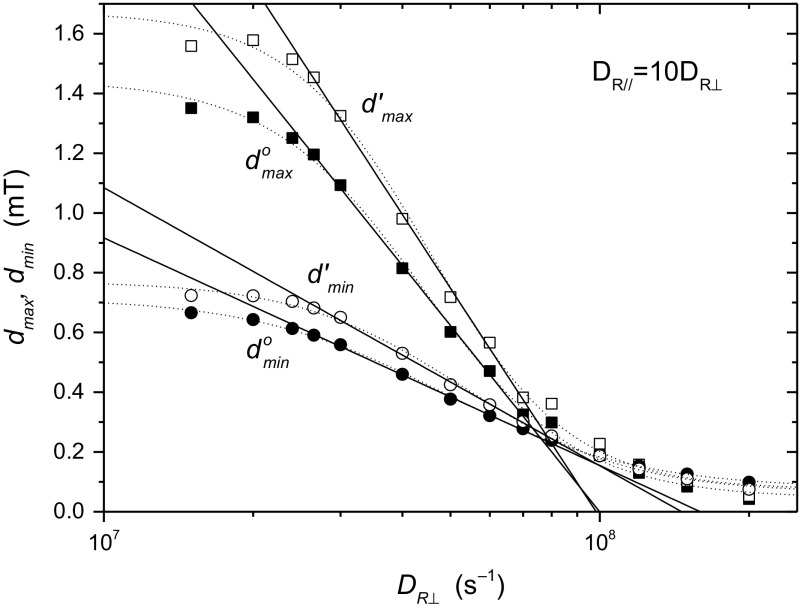
Dependence on rotational diffusion coefficient $D_{{{\text{R}} \bot }} ( = 0.1D_{{{\text{R}}//}} )$ of linear regression parameters for the line shifts $d_{\hbox{max} } = A_{\hbox{max} } - \left\langle {A_{//} } \right\rangle$, and $d_{\hbox{min} } = \left\langle {A_{ \bot } } \right\rangle - A_{\hbox{min} }$, as a function of $S_{zz}$ (see Fig. 2). *Solid lines* are linear regressions over the range $D_{{{\text{R}} \bot }} = (2.65 - 8.0) \times 10^{7} {\text{ s}}^{ - 1}$ for the logarithmic *x*-axis. *Dotted lines* are fits of the logistic equation (see Table 1)

Values of the parameters, $d_{\hbox{max} }^{\text{o}}$, $d^{\prime}_{\hbox{max} }$ and $d_{\hbox{min} }^{\text{o}}$, $d^{\prime}_{\hbox{min} }$, for different values of $D_{{{\text{R}} \bot }}$ are obtained by linear regression. Figure 3 shows the dependence of these calibration parameters in Eqs. 5 and 6 on diffusion coefficient. Note that the $D_{{{\text{R}} \bot }}$-axis is logarithmic. We see that the dependence for all four parameters is approximately logarithmic over the range $D_{{{\text{R}} \bot }} = (2.65 - 8.0) \times 10^{7} {\text{ s}}^{ - 1}$, in the slow-motion regime. This dependence is given by the solid lines. In the fast-motion region $(D_{{{\text{R}} \bot }} \ge 1 \times 10^{8} {\text{ s}}^{ - 1} )$, the linear dependence of $d_{\hbox{max} }^{{}}$ on order parameter is slight and restricted to the range $S_{zz} = 0.5 - 0.8$. The dotted lines in Fig. 3 are fits of the logistic equation: $y = {{(c_{1} - c_{2} )} \mathord{\left/ {\vphantom {{(c_{1} - c_{2} )} {[1 + (x/x_{o} )^{p} ] + c_{2} }}} \right. \kern-0pt} {[1 + (x/x_{o} )^{p} ] + c_{2} }}$, with logarithmic *x*-axis. These provide fits over a wider range of diffusion coefficient, but with lower precision. We return to this type of fit later.

Substituting from Eqs. 5 and 6 into Eq. 3, we get the following calibration for the corrected order parameter:

7 $$S_{zz} = \frac{{\left( {A_{\hbox{max} } - A_{\hbox{min} } } \right) - \left( {d_{\hbox{max} }^{o} + d_{\hbox{min} }^{o} } \right)}}{{\Delta A - \left( {d^{\prime}_{\hbox{max} } - d^{\prime}_{\hbox{min} } } \right)}}$$

in terms of the measurables $A_{\hbox{max} }$ and $A_{\hbox{min} }$. We therefore write Eq. 7 as:

8 $$S_{zz} = s_{1} \times \left( {A_{\hbox{max} } - A_{\hbox{min} } } \right) - s_{o}$$

where the calibration parameters $s_{1}$ and $s_{\text{o}}$ are given for different values of $D_{{{\text{R}} \bot }}$ in Table 1. Logistic fits of the dependence on diffusion coefficient (cf. dotted lines in Fig. 3), over the full range $D_{{{\text{R}} \bot }} = (1.5 - 20.0) \times 10^{7} {\text{ s}}^{ - 1}$, are given in the footnote to Table 1. We get higher precision, but over a more limited range, by using linear regression with logarithmic $D_{{{\text{R}} \bot }}$-axis (cf. solid lines in Fig. 3):

9 $$s_{1} \, = 2.828 - 0.313 \times \log_{10} D_{{{\text{R}} \bot }} ({\text{s}}^{ - 1} )$$

10 $$s_{o} = 13.038 - 1.637 \times \log_{10} D_{{{\text{R}} \bot }} ({\text{s}}^{ - 1} )$$

over the range $D_{{{\text{R}} \bot }} = (2.65 - 8.0) \times 10^{7} {\text{ s}}^{ - 1}$. When in the fast regime, $A_{\hbox{max} } = \left\langle {A_{//} } \right\rangle$ over the range $S_{zz} = 0. 1- 0. 4$, and the corresponding calibration becomes:

**Table 1:** Calibration parameters in Eqs. 8 and 13 for order parameter and isotropic hyperfine coupling, for different diffusion coefficients $D_{R \bot } ( = 0.1 \times D_{R//} )$

$D_{{{\text{R}} \bot }}$ (s^−1^)	$s_{1}$ (mT^−1^)	$s_{\text{o}}$	$f_{\hbox{max} }$	$f_{\hbox{min} }$	$\delta a_{\text{o}}$ (mT)
1.5 × 10^7^	0.529	1.067	1.020	1.980	−0.046
2.0 × 10^7^	0.535	1.050	1.024	1.976	−0.058
2.4 × 10^7^	0.522	0.973	1.019	1.981	−0.043
2.65 × 10^7^	0.512	0.914	1.015	1.985	−0.032
3.0 × 10^7^	0.488	0.805	1.004	1.996	0.002
4.0 × 10^7^	0.440	0.560	0.988	2.012	0.050
5.0 × 10^7^	0.411	0.402	0.982	2.018	0.068
6.0 × 10^7^	0.397	0.315	0.980	2.020	0.073
7.0 × 10^7^	0.378	0.228	0.972	2.028	0.093
8.0 × 10^7^	0.382	0.205	0.982	2.018	0.070
1.0 × 10^8^	0.373	0.140	0.982	2.018	0.069
1.2 × 10^8^	0.369	0.105	0.984	2.016	0.063
1.5 × 10^8^	0.366	0.077	0.986	2.014	0.059
	(0.353	0.044	0.974	2.026	0.087)^a^
2.0 × 10^8^	0.364	0.051	0.988	2.012	0.053
	(0.357	0.035	0.982	2.018	0.067)^a^

Empirical logistic fits

$$a_{1}^{{}} ({\text{mT}}^{ - 1} ) = \frac{0.174}{{1 + \left( {{{D_{\text{R}} } \mathord{\left/ {\vphantom {{D_{\text{R}} } {3.780 \times 10^{7} }}} \right. \kern-0pt} {3.780 \times 10^{7} }}} \right)^{4.011} }} + 0.368$$

$$a_{0}^{{}} = \frac{1.092}{{1 + \left( {{{D_{\text{R}} } \mathord{\left/ {\vphantom {{D_{\text{R}} } {3.892 \times 10^{7} }}} \right. \kern-0pt} {3.892 \times 10^{7} }}} \right)^{3.005} }} + 0.067$$

$$f_{\hbox{max} }^{{}} = 0.982 + \frac{0.04}{{1 + \left( {{{D_{\text{R}} } \mathord{\left/ {\vphantom {{D_{\text{R}} } {3.12 \times 10^{7} }}} \right. \kern-0pt} {3.12 \times 10^{7} }}} \right)^{8.74} }}$$

$$f_{\hbox{min} }^{{}} = 2.018 - \frac{0.04}{{1 + \left( {{{D_{\text{R}} } \mathord{\left/ {\vphantom {{D_{\text{R}} } {3.12 \times 10^{7} }}} \right. \kern-0pt} {3.12 \times 10^{7} }}} \right)^{8.74} }}$$

$$\delta a_{o}^{{}} = 0.068 - \frac{0.122}{{1 + \left( {{{D_{\text{R}} } \mathord{\left/ {\vphantom {{D_{\text{R}} } {3.12 \times 10^{7} }}} \right. \kern-0pt} {3.12 \times 10^{7} }}} \right)^{8.68} }}$$

^a^Fits with $$d_{\hbox{max} }^{\text{o}} = 0$$, $$d_{\hbox{max} }^{\prime } = 0$$

11 $$S_{zz} = 0.355 \times \left( {A_{\hbox{max} } - A_{\hbox{min} } } \right) - 0.040.$$

For comparison, previous corrections require subtraction of 0.03–0.05 for fast motion [1–3].

We also get corrected values of the isotropic hyperfine coupling, $a_{o}^{{}} = \tfrac{1}{3}(\left\langle {A_{//} } \right\rangle + 2\left\langle {A_{ \bot } } \right\rangle )$, from Eqs. 5 and 6:

12 $$a_{o}^{{}} = \tfrac{1}{3}\left( {A_{\hbox{max} } + 2A_{\hbox{min} } } \right) - \tfrac{1}{3}\left( {d_{\hbox{max} }^{o} - 2d_{\hbox{min} }^{o} } \right) + \tfrac{1}{3}\left( {d^{\prime}_{\hbox{max} } - 2d^{\prime}_{\hbox{min} } } \right) \times S_{zz}.$$

This depends on $S_{zz}$, which we substitute from Eq. 7 (or 8) to give the following general form:

13 $$a_{o}^{{}} = \tfrac{1}{3}\left( {f_{\hbox{max} } A_{\hbox{max} } + f_{\hbox{min} } A_{\hbox{min} } } \right) + \delta a_{o}$$

where the calibration parameters $f_{\hbox{max} }$, $f_{\hbox{min} }$ and $\delta a_{o}$ are given for different values of $D_{{{\text{R}} \bot }}$ in Table 1. Using linear regression with logarithmic $D_{{{\text{R}} \bot }}$-axis (cf. Fig. 3), we get the approximate dependence of the calibration parameters:

14 $$f_{\hbox{max} } \, = 1.713 - 0.095 \times \log_{10} D_{{{\text{R}} \bot }} ({\text{s}}^{ - 1} )$$

15 $$f_{\hbox{min} } = 1.287 + 0.095 \times \log_{10} D_{{{\text{R}} \bot }} ({\text{s}}^{ - 1} )$$

16 $$\delta a_{o} ({\text{mT}}) = - 2.075 + 0.277 \times \log_{10} D_{{{\text{R}} \bot }} ({\text{s}}^{ - 1} )$$

over the range $D_{{{\text{R}} \bot }} = (2.65 - 8.0) \times 10^{7} {\text{ s}}^{ - 1}$. Again, logistic fits over the full range of $D_{{{\text{R}} \bot }}$ are included in the footnote to Table 1. Note that corrections to $a_{\text{o}}^{{}}$ for slow motion are smaller than those to the order parameter, because the opposite shifts in $A_{\hbox{max} }$ and $A_{\hbox{min} }$ compensate. When in the fast regime, the calibration corresponding to Eq. 11 becomes:

17 $$a_{o}^{{}} ({\text{mT}}) = \tfrac{1}{3}\left( {0.978A_{\hbox{max} } + 2.022A_{\hbox{min} } } \right) + 0.077.$$

For comparison, previous treatments make an additive correction of 0.05–0.09 mT for fast motion [1–3]

When calculating the order parameter, we use the isotropic hyperfine coupling to correct for differences between the hyperfine tensor of the experimental sample and that used to get $\Delta A$ for Eq. 3. The spin-Hamiltonian tensors used here, with $\Delta A$ = 2.74 mT (see Fig. 1), correspond to the MTSSL spin used in site-directed spin labelling (see, e.g., [22]). They correspond reasonably closely with those for DOXYL spin labels, which also have a five-membered ring. To make the correction, we simply scale $\left( {A_{\hbox{max} } - A_{\hbox{min} } } \right)$ in Eq. 8 or 11 by the factor: ${{\tfrac{1}{3}\left( {A_{xx} + A_{yy} + A_{zz} } \right)} \mathord{\left/ {\vphantom {{\tfrac{1}{3}\left( {A_{xx} + A_{yy} + A_{zz} } \right)} {a_{o}^{{}} = }}} \right. \kern-0pt} {a_{o}^{{}} = }}{{1.513{\text{ mT}}} \mathord{\left/ {\vphantom {{1.513{\text{ mT}}} {a_{o}^{{}} }}} \right. \kern-0pt} {a_{o}^{{}} }}$, where $a_{\text{o}}^{{}}$ comes from Eq. 13 or 17, respectively. This corrects for differences in environmental polarity, and to a first approximation for differences between nitroxide structures (e.g., TOAC spin labels with the six-membered piperidine ring).

**Fig. 4:**
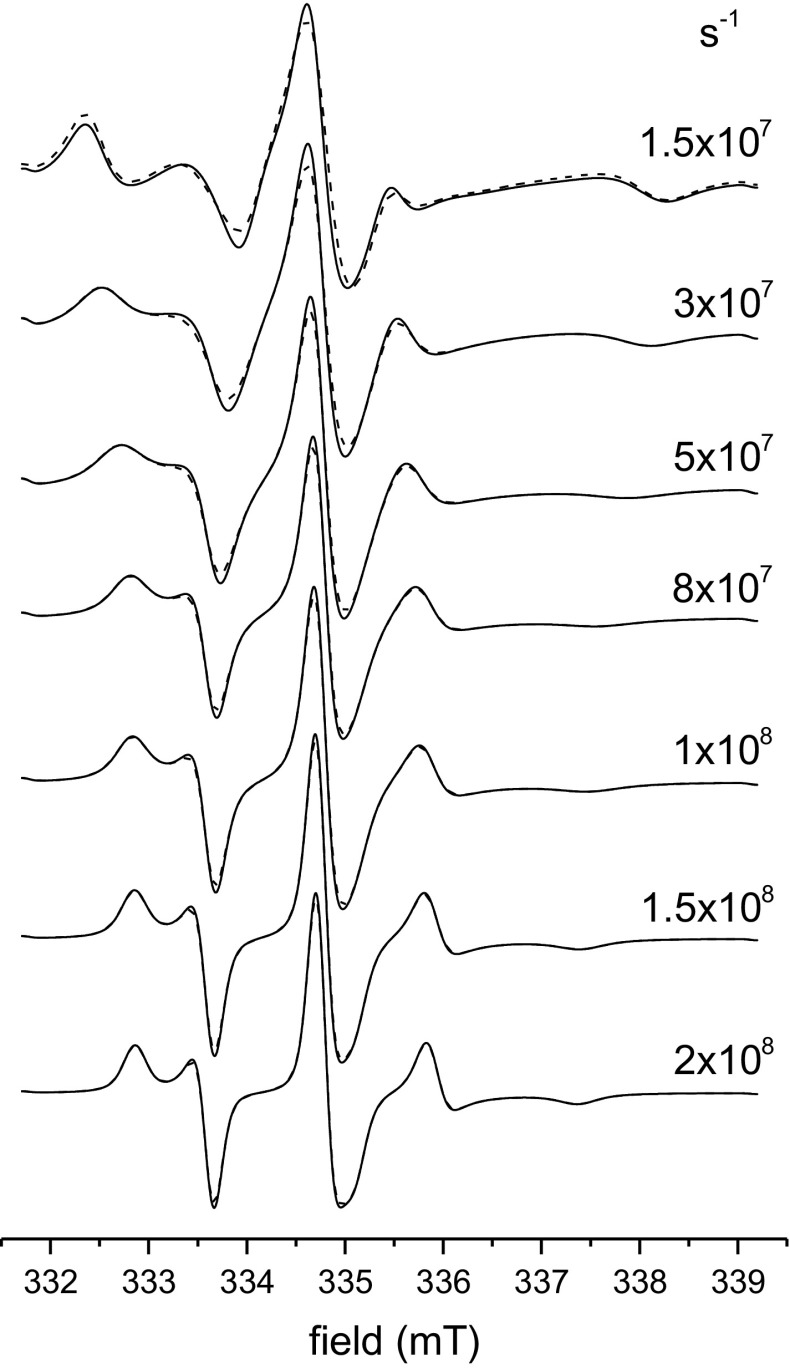
Stochastic-Liouville simulations of ^14^N-nitroxide 9.4-GHz EPR powder spectra, for different values of the rotational diffusion coefficient $D_{{{\text{R}} \bot }} = 0.1D_{{{\text{R}}//}}$ (*solid lines*) and $D_{{{\text{R}} \bot }} = D_{{{\text{R}}//}}$ (*dashed lines*), as indicated, and fixed order parameter $S_{zz} = 0.4$. Simulation parameters otherwise as in Fig. 1

The calibration parameters in Table 1 are obtained from simulations for fast axial diffusion: $D_{{{\text{R}}//}} = 10D_{{{\text{R}} \bot }}$, corresponding to a prolate rigid rotator with axial ratio ${a \mathord{\left/ {\vphantom {a b}} \right. \kern-0pt} b} \approx 6.5$ [23]. For segmental rotation, however, the elements of the diffusion tensor are more likely to be equal. Figure 4 compares spectra simulated for $D_{{{\text{R}}//}} = 10D_{{{\text{R}} \bot }}$ (solid lines) with those for $D_{{{\text{R}}//}} = D_{{{\text{R}} \bot }}$ (dashed lines), where the order parameter is now $S_{zz} = 0. 4$; in each case the value of $D_{{{\text{R}} \bot }}$ is the same. We see some differences in line shape for slow motion, but not in the fast motional regime. Table 2 gives the calibration parameters for order parameter and isotropic hyperfine coupling when $D_{{{\text{R}}//}} = D_{{{\text{R}} \bot }}$. Only in the slow motion regime do these differ from those in Table 1, and mostly the differences are not large. We conclude from this that calibration parameters depend markedly on $D_{{{\text{R}} \bot }}$ in the slow motion regime, but little on the rotational anisotropy ${{D_{{{\text{R}}//}} } \mathord{\left/ {\vphantom {{D_{{{\text{R}}//}} } {D_{{{\text{R}} \bot }} }}} \right. \kern-0pt} {D_{{{\text{R}} \bot }} }}$.

**Table 2:** Calibration parameters in Eqs. 8 and 13 for order parameter and isotropic hyperfine coupling, for different diffusion coefficients $D_{\text{R}} \equiv D_{{{\text{R}} \bot }} ( = D_{{{\text{R}}//}} )$

$D_{\text{R}}$ (s^−1^)	$s_{1}$ (mT^−1^)	$s_{o}$	$f_{\hbox{max} }$	$f_{\hbox{min} }$	$\delta a_{o}$ (mT)
1.5 × 10^7^	0.544	1.078	1.039	1.961	−0.105
2.0 × 10^7^	0.540	1.052	1.031	1.969	−0.081
2.4 × 10^7^	0.527	0.975	1.026	1.975	−0.064
2.65 × 10^7^	0.516	0.916	1.021	1.980	−0.049
3.0 × 10^7^	0.493	0.814	1.010	1.990	−0.016
4.0 × 10^7^	0.446	0.562	0.998	2.002	0.022
5.0 × 10^7^	0.427	0.438	0.995	2.005	0.034
6.0 × 10^7^	0.404	0.328	0.986	2.014	0.058
7.0 × 10^7^	0.380	0.229	0.974	2.026	0.087
8.0 × 10^7^	0.368	0.170	0.970	2.030	0.097
1.0 × 10^8^	0.374	0.144	0.983	2.017	0.066
1.2 × 10^8^	0.369	0.105	0.984	2.016	0.064
1.5 × 10^8^	0.367	0.076	0.987	2.013	0.057
	(0.353	0.044	0.975	2.025	0.086)^a^
2.0 × 10^8^	0.364	0.051	0.988	2.012	0.053
	(0.357	0.035	0.982	2.018	0.067)^a^

^a^Fits with $$d_{\hbox{max} }^{\text{o}} = 0$$, $$d_{\hbox{max} }^{\prime } = 0$$

## Discussion and Conclusions

Experience shows that spin-label spectra from systems exhibiting partial molecular order frequently contain components that are in the slow motional regime for nitroxide EPR. In such cases, we must make corrections to both $A_{\hbox{max} }$ and $A_{\hbox{min} }$, which we obtain from the canonical turning points in pseudo-powder spectra from non-aligned samples, when calculating orientational order parameters and isotropic hyperfine couplings. Because of partial cancellation in the latter case, corrections are more important for $S_{zz}$ than for $a_{\text{o}}$. A previous treatment with an oversimplified slow-motion model gave no correction for $A_{\hbox{max} }$ because a near rigid-limit value of $D_{{{\text{R}} \bot }}$ was used to emulate a powder distribution [3].

To calculate order parameters for slow motion, we must know the diffusion tensor element $D_{{{\text{R}} \bot }}$ that characterizes angular excursions of the molecular long axis (to which the nitroxide *z*-axis is assumed approximately parallel). This requires simulation results for at least one spectrum in an experimental series. The spectra in Figs. 1 and 4, which correspond to two different order parameters, can be a helpful guide here. We then get order parameter and isotropic coupling constant from Eqs. 8 and 13, respectively, by using the appropriate calibration constants listed in Table 1 (or Table 2). Alternatively, the calibration constants come from Eqs. 9, 10, 14–16 for $D_{{{\text{R}} \bot }} = (2.65 - 8.0) \times 10^{7} {\text{ s}}^{ - 1}$, or less precisely outside this range from the logistic fits listed at the bottom of Table 1. As we see from Fig. 4, the value of $D_{{{\text{R}} \bot }}$ is crucial, whereas the faster rotation around the molecular axis, $D_{{{\text{R}}//}} ( \ge D_{{{\text{R}} \bot }} )$, is relatively unimportant.

The calibrations given here are for partially averaged powder spectra that display well defined axial anisotropy. For this, the nitroxide *z*-axis must lie close to the long molecular axis that is being ordered, because this is the principal axis for the N-hyperfine anisotropy. If the nitroxide *z*-axis is inclined at angle $\theta_{\text{NO}}$ to the molecular axis, we know from the spherical harmonic addition theorem that the order parameter measured by EPR is given by: $S_{zz} = {\text{P}}_{2} (\cos \theta_{\text{NO}} ) \times S_{\text{mol}}$, where $S_{\text{mol}}$ is the order parameter of the long molecular axis, and ${\text{P}}_{2} (\cos \theta_{\text{NO}} )$ is a second rank Legendre polynomial. As already mentioned, practical examples of spin labels with the appropriately oriented nitroxide axes are DOXYL-labelled hydrocarbon chains, and TOAC in peptide helices. Complications arise with lipid chains because motion of the long molecular axis often lies in the slow regime [24], but rotational isomerism of the chain segments is much faster [25, 26]. In this case, the effective hyperfine anisotropy is reduced from $\Delta A$ to $\Delta A \times S_{t - g}$ by rapid *trans*-*gauche* isomerism in the chain (cf. Eqs. 1 and 2). Order-parameter calculations for the slow long-axis motion are then made using this reduced anisotropy. Multifrequency EPR is helpful in separating fast and slow components of spin-label motion. For instance, the slower long-axis motion of lipid chains in phospholipid membranes is driven into the rigid limit at 94-GHz EPR frequency, and the high field spin-label spectrum reflects motional averaging $S_{t - g}$ by *trans*-*gauche* isomerism alone [27–29].

Although the slow-motion order parameter calibrations require an estimate of the diffusion coefficient, they offer significant improvements over those based purely on motional narrowing theory. Even when a full spectral simulation study is to be attempted, the calibrations given here are a useful starting point to establish initial simulation parameters.

It goes without saying that the present corrections apply only to those pseudo-powder spectra which show a well-defined inner hyperfine splitting, $2A_{\hbox{min} }$. Although this accounts for a major class of spectra from important spin labels, there are numerous cases when this does not apply, e.g., polymeric liquid crystals and glasses (see for instance [30]).
